# West Nile Virus: Immunity and Pathogenesis

**DOI:** 10.3390/v3060811

**Published:** 2011-06-15

**Authors:** Stephanie M. Lim, Penelope Koraka, Albert D.M.E. Osterhaus, Byron E.E. Martina

**Affiliations:** Department of Virology, Erasmus MC, P.O. Box 2040, 3000 CA Rotterdam, The Netherlands; E-Mails: s.lim@erasmusmc.nl (S.M.L.); p.koraka@erasmusmc.nl (P.K.); a.osterhaus@erasmusmc.nl (A.D.M.E.O.)

**Keywords:** West Nile virus, pathogenesis, central nervous system, neuroinvasion

## Abstract

West Nile virus (WNV) is a neurotropic, arthropod-borne flavivirus that is maintained in an enzootic cycle between mosquitoes and birds, but can also infect and cause disease in horses and humans. WNV is endemic in parts of Africa, Europe, the Middle East, and Asia, and since 1999 has spread to North America, Mexico, South America, and the Caribbean. WNV infects the central nervous system (CNS) and can cause severe disease in a small minority of infected humans, mostly immunocompromised or the elderly. This review discusses some of the mechanisms by which the immune system can limit dissemination of WNV infection and elaborates on the mechanisms involved in pathogenesis. Reasons for susceptibility to WNV-associated neuroinvasive disease in less than 1% of cases remain unexplained, but one favored hypothesis is that the involvement of the CNS is associated with a weak immune response allowing robust WNV replication in the periphery and spread of the virus to the CNS.

## 1. Introduction

West Nile virus (WNV) is a neurotropic, arthropod-borne flavivirus with a single-stranded positive-sense RNA genome. WNV is maintained in an enzootic cycle between mosquitoes and birds, but can also infect and cause disease in horses and humans, which serve as incidental dead-end hosts. WNV is endemic in parts of Africa, Europe, the Middle East, and Asia [1], and following its emergence in the United States in 1999 it has rapidly spread across North America, and has recently been reported in Mexico, South America, and the Caribbean [2–4]. Currently, no specific therapy or vaccine has been approved for use against WNV infection in humans.

Epidemiological studies indicated that the frequency and severity of clinical illness increases with age [5–7]. Infection with WNV remains asymptomatic in the majority of cases or results in West Nile fever (a mild flu-like illness) in approximately 20 to 30% of infected cases [7–9]. Symptoms are of sudden onset and may include malaise, eye pain, headache, myalgia, gastro-intestinal discomfort and rash [8,10–13]. A small percentage of cases may develop encephalitis, meningitis, or acute flaccid paralysis (AFP) [6,7,13], and long-term neurological sequelae are common in more than 50% of these cases [14–18]. Disease manifestation is explained by neuronal damage in several regions of the brain. The fatality rate for hospitalized encephalitic cases is approximately 10%, with increased risk for patients with compromised immune systems, advanced age and with underlying conditions such as diabetes mellitus [19].

Despite the significance of central nervous system (CNS) pathology in severe disease, the mechanisms by which WNV and other encephalitic flaviviruses cause neuronal injury *in vivo* have not been completely elucidated. The increased risk for immunosuppressed patients seems to suggest that an intact immune system is essential for the control of WNV infection. Although peripheral immune responses to WNV can prevent encephalitis, up to 40% of immunocompetent animals infected with a virulent WNV strain develop lethal neuroinvasive disease [20,21]. In these cases the pathologic effect of the immune system cannot be excluded.

It is interesting to note that several other flaviviruses are known to cause neuroinvasive disease, but little is known about the pathogenic mechanisms. There is a need for comparative studies between these different viruses. Studies of flaviviruses that rarely cause neuroinvasive disease may also contribute to a better understanding of the mechanisms involved in viral entry in the brain. For instance, infection with dengue virus (DENV), one of the most important arboviral diseases of humans, may result in severe systemic disease, manifested as hemorrhagic or shock syndrome [22]. Although DENV has been considered to be a non-neurotropic virus, recent observations indicate that the clinical profile of DENV infection is changing, with neurological manifestations becoming more frequent [23,24]. The neuropathogenesis of DENV, and the contribution of viral and host factors to neuroinvasive disease are not well understood. Barker *et al*. [25] noticed that flaviviruses that cause hemorrhagic disease have asparagine (Asn) at position 67 of the E protein, corresponding to the glycosylation site, whereas neurotropic viruses contain Asp at this position. Experiments should be designed to study these associations.

This review will discuss the immune responses in the periphery and brain necessary for the control of WNV infection, as well as the particular responses that may be involved in the development of encephalitis.

## 2. Tropism

It is believed that after a mosquito bite WNV infects keratinocytes [26] and Langerhans cells, which migrate to regional lymph nodes where initial replication occurs [27–31]. WNV then spreads systemically to visceral organs, such as the kidney and spleen, where a second round of replication takes place, presumably in epithelium cells and macrophages, respectively [32]. Depending on the level of viremia WNV can cross the blood-brain barrier (BBB) into the brain and cause meningo-encephalitis. This is similar to what has been observed for other neurotropic viruses, such as Saint-Louis encephalitis virus in mice [33], where the probability of neuroinvasion appeared to correlate with the level and duration of viremia. The envelope (E) glycoprotein of WNV has been implicated in neuroinvasiveness, particularly domain III of the protein, which constitutes the receptor binding domain [34–36]. Several mechanisms have been proposed for WNV entry into the CNS: (i) infection or passive transport through the endothelium or choroid plexus epithelial cells [37], (ii) infection of olfactory neurons and spread to the olfactory bulb [38], (iii) a “Trojan horse” mechanism in which the virus is transported by infected immune cells trafficking to the CNS [39], and (iv) direct axonal retrograde transport from infected peripheral neurons [40–42].

**Figure 1.**
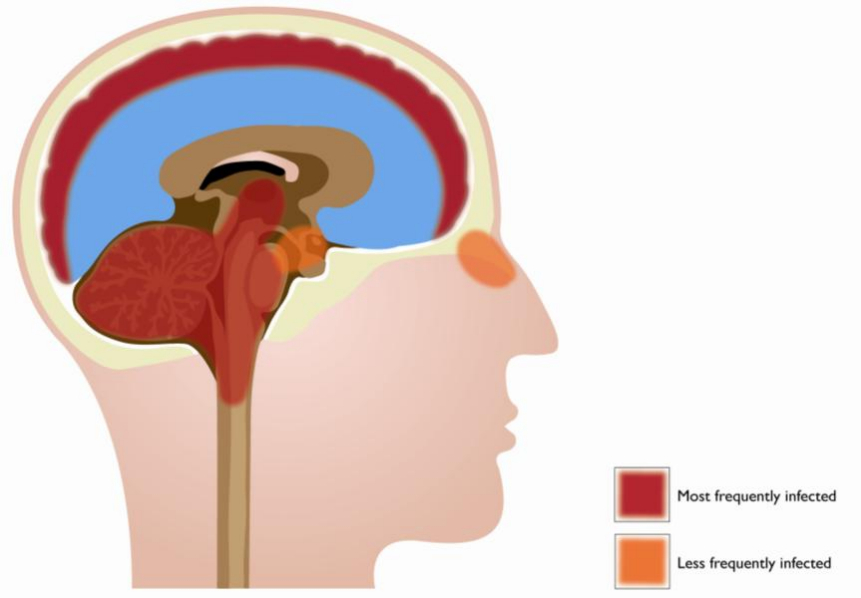
Frequency of infection of several regions of the human brain by West Nile virus. The areas most often infected by WNV include: the cerebral cortex, thalamus, basal ganglia, brainstem, cerebellum, and spinal cord (anterior horn) (indicated in dark red). Infection has less frequently been found in the olfactory bulb and hippocampus (indicated in orange).

In humans, WNV is most often detected in neurons in the cerebral cortex, thalamus, brainstem, basal ganglia, cerebellum, and spinal cord (mainly anterior horn), and in some cases, infection has been detected in the olfactory bulb and hippocampus (Figure 1) [43]. WNV has been detected in the same regions of the brain of experimentally infected mice as in humans, indicating a similar tropism of WNV in humans and mice. The synchronous appearance of virus at many sites in the brain and spinal cord suggests that haematogenous spread is an obvious way of entry into the CNS. Histological analysis of samples from fatal human cases also provided evidence of gliosis, indicating involvement of microglial cells and astrocytes during WNV infection. *In vitro* experiments have shown that WNV can infect primary neurons, human and mouse neuroblastoma cells, cortical astrocytes (HBCA), brain microvascular endothelial (HBMVE) cells [44,45], and oligodendrocytes [46–49], while infection of microglia resulted in low viral yield [44]. So far, animal experiments have only shown infection of neurons by WNV [50–53] and have provided limited evidence of *in vivo* glial cell infection.

## 3. Adaptive Immune Response Protects against Severe WNV Infection

It has been recognized that the elderly and immunocompromised are especially at risk for disseminated WNV infection and for developing fatal encephalitis. Although the exact immunological basis for the increased susceptibility in the elderly remains unclear, experiments with mice have begun to elucidate the role of the different components of the innate and adaptive immune response in controlling infection, in particular the role of immunoglobulin M (IgM), CD4+ and CD8+ T cells. It is believed that changes in both innate and adaptive immune function converge in the reduced response to vaccination and protection against infection in the elderly [54]. For instance, the decline in thymic output of naïve T cells diminishes responses to novel antigens, such as WNV, while clonal expansions leading to defects in the T cell repertoire are associated with blunted responses of memory T cells to conserved epitopes of the influenza virus [54].

Use of a well-characterized mouse model of WNV infection, which in many respects resembles the human disease, showed that mice deficient in the production of secreted IgM (sIgM), but still capable of expressing surface IgM were vulnerable to lethal infection, even after inoculation with low doses of WNV [55]. sIgM−/− mice developed higher levels of infectious virus in sera compared to wild-type animals. This enhanced viremia correlated with higher WNV burdens in the CNS. Consistently, passive transfer of polyclonal anti-WNV IgM or IgG protected sIgM−/− mice against mortality, while administration of comparable amounts of a non-neutralizing monoclonal anti-WNV IgM did not provide any protection. Overall, these results indicate that the induction of a specific, neutralizing IgM response early in the course of WNV infection limits viremia and dissemination of virus into the CNS, resulting in protection against lethal infection. Whether the kinetics of the IgM response to WNV differs between young and the elderly and how it might affect susceptibility to severe WNV infection in humans is not clear.

Furthermore, it has also been demonstrated that mice with a genetic or acquired deficiency in CD4+ T lymphocytes display protracted WNV infection in the CNS, leading to uniform lethality by 50 days after infection [56]. Mice that survived past day 10 had high-level persistent infection in the CNS compared to wild-type mice, even up to 45 days after infection. WNV-specific IgM levels decreased about 20-fold at day 15 post-infection in CD4-deficient mice and IgG levels were about 100- to 1,000-fold lower throughout the course of infection compared to wild-type mice. Furthermore, WNV-specific CD8+ T-cell activation and trafficking to the CNS were markedly compromised at day 15. These results suggest that the main protective role of CD4+ T cells during primary infection of WNV is to assist in antibody responses and to sustain WNV-specific CD8+ T cell responses in the CNS that enable viral clearance.

CD8+ T cells have been shown to directly play a role in controlling WNV infection and preventing severe disease. When mice lacking CD8+ T cells or classical Ia major histocompatibility complex (MHC) antigens were infected with a virulent WNV isolate, high viral titers were recovered from the CNS and increased mortality rates were recorded [57]. In contrast, absence of CD8+ T cells did not affect the quantitative antibody response and did not alter the kinetics or magnitude of viremia during primary infection. Interestingly, infectious virus could still be recovered from the CNS of CD8+ T cell-deficient mice that survived initial WNV challenge for several weeks. It is interesting however, that in spite of the normal quantitative antibody response, WNV was still able to enter the brain, even though it has been shown that IgM and IgG are important in preventing dissemination of WNV into the CNS [55]. These experiments collectively suggest that WNV-specific antibodies are responsible for reducing viremia and preventing development of severe disease, while CD8+ T cells play an important function in clearing infection from tissues and preventing viral persistence. Whether antibodies can prevent neuroinvasion remains to be determined.

## 4. Crossing the Blood-Brain-Barrier (BBB)

Inflammation has been functionally defined as the host response to injury, meant to eliminate the cause of damage and promote tissue healing. However, several studies have incriminated inflammation in the pathogenesis of numerous chronic diseases such as cancer, diabetes, or neurodegenerative conditions. The limited regenerative capacity of neurons makes the CNS particularly susceptible to damage mediated by inflammation. For a long time it was assumed that the CNS is protected from extensive immunological responses due to restricted leukocyte trafficking from the periphery to the CNS, a process erroneously referred to as “immune-privilege”. Initially, CNS immune-privilege was attributed to CNS isolation from the immune system by the BBB, the lack of draining lymphatics, and the apparent immune-incompetence of microglia, the resident CNS macrophages. However, this view has been revisited based on data showing that peripheral immune cells can cross the intact BBB.

The CNS is populated by three different glial cell populations: (1) astrocytes, (2) microglia, and (3) oligodendrocytes. Glial cells are believed to form the immune system in the brain. Astrocytes are star-shaped and constitute the most abundant cell type found in the CNS; it has been suggested that there are approximately 10-times more astrocytes than neurons [58,59], although a ratio of only 1.4 [60] has also been indicated. Astrocytes have a number of important functions in the brain homeostasis, including maintenance of the BBB, regulation of neuronal blood flow, regulation of the extracellular potassium balance, providing metabolic support to neurons, and stimulation of myelin formation by oligodendrocytes. Although astrocytes can produce acute-phase proteins and some pro-inflammatory cytokines, in general they have limited innate immune properties. Astrocytes contribute to the maintenance of the BBB by creating a network of foot processes called the glia limitans. In this regard, astrocytes lie at the gateway to the CNS parenchyma and play a crucial role in controlling leukocyte influx.

Microglia are the resident macrophages of the brain and comprise 10–20% of all glial cells. Their main function is to monitor and maintain neuronal health. They are very sensitive and become readily activated by most neuropathologic conditions, including inflammatory disease and neuronal injury. When activated, microglia display distinct functional plasticity, such as changes in cell morphology, cell number, cell surface receptor expression, and production of growth factors and cytokines. It has been shown that microglia are immunocompetent, but differ from macrophages and dendritic cells in their ability to orchestrate neuroprotective lymphocyte responses. In addition, CNS neurons and glial cells have been shown to be capable of actively regulating macrophage and lymphocyte responses [61].

Oligodendrocytes are a type of neuroglia found in the CNS of vertebrates and invertebrates. Their function is to produce myelin, which acts as an insulating sheath on the axons of nerve fibers. Oligodendrocytes can be distinguished from astrocytes by their greater density of both the cytoplasm and the nucleus, the absence of fibrils and glycogen in the cytoplasm, and the large numbers of microtubules in the processes. A single oligodendrocyte can extend its processes to approximately 50 axons, wrapping around approximately 1 μm of myelin sheath of each axon.

**Figure 2.**
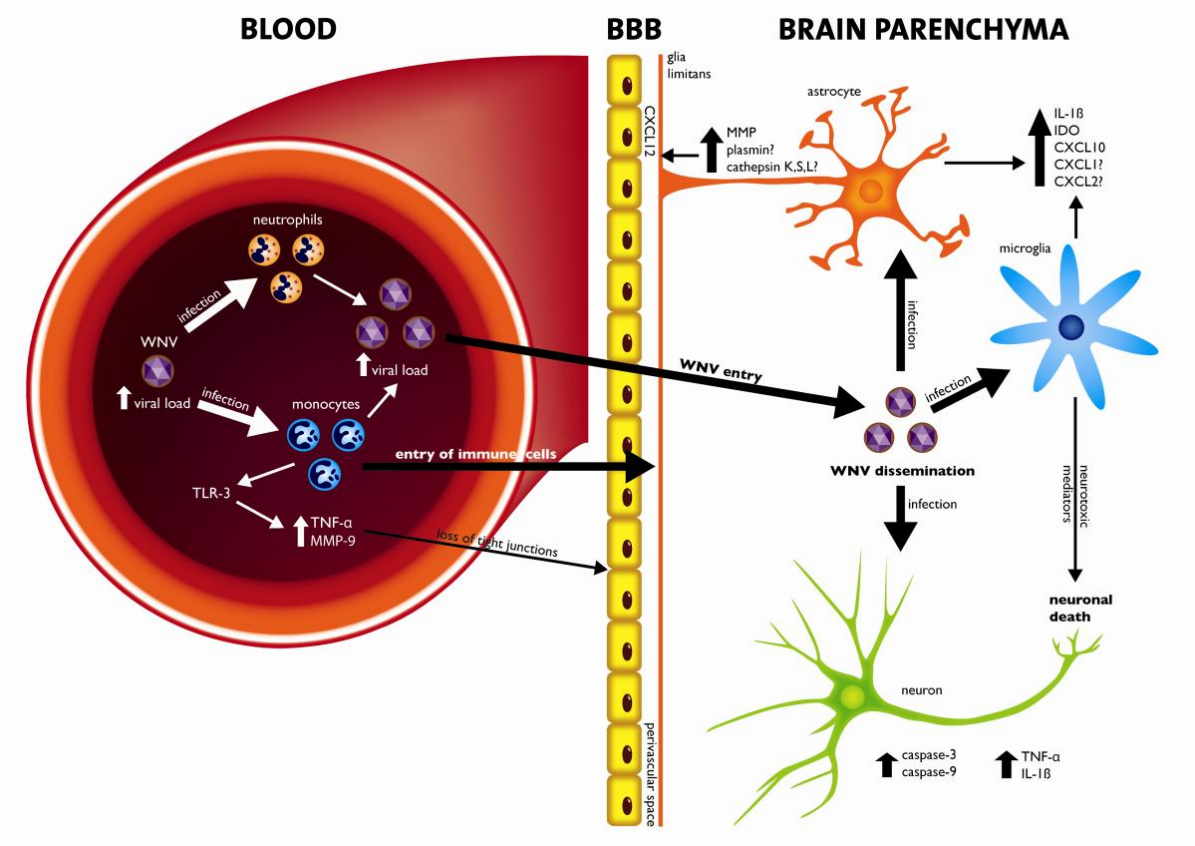
Regulation of Blood-brain barrier permeability, entry of WNV in the brain and factors involved in the pathogenesis of WNV-induced neuroinvasisve disease. Following inoculation of WNV in the dermis, virus infects and replicates in cells of the mononuclear lineage and neutrophils. Monocytes and neutrophils act as reservoirs for viral replication and dissemination, resulting in an increase in viral load. Recognition of WNV replication by TLR3 in monocytes leads to production of TNF-α and MMP-9 in a dose-dependent manner, resulting in loss of tight junctions, which allows entry of WNV and immune cells into the brain. High viral loads facilitate entry and dissemination of WNV into the brain. Expression of CXCL12 plays an important role in the retention of immune cells in the perivascular spaces of the CNS. Production of MMPs, cathepsins and plasmin by activated/infected monocytes and glial cells are probably involved in migration of cells from the perivascular space into the brain parenchyma. Furthermore, infected glial cells and neurons release neurotoxic mediators, leading to neuronal death. Neuronal death is mediated for a great part via the caspase-9 and caspase-3 pathway, which is dependent on the capsid of WNV. WNV: West Nile virus; TNF-α: Tumor necrosis factor-α; MMP: Matrix metalloproteinases; TLR: Toll-like receptor; CXCL: chemokine; IL: Interleukin; BBB: Blood-brain barrier; glial limitans: a network of foot processes of astrocytes.

In mice, WNV crosses the BBB and infects the CNS after the peak viremia (around day 3) [62,63]. Several studies showed that immune responses in the brain are necessary to clear the infection. How exactly recruitment of leukocytes from blood into the brain parenchyma is regulated is however not completely understood. Recent data suggest that leukocytes have to pass two barriers in order to arrive in the brain parenchyma: the endothelial cell vascular wall and the glial limitans, collectively referred to as BBB (Figure 2). After transmigration of the vascular wall, the majority of infiltrating leukocytes are retained in the perivascular space, but the factors that regulate this process have not yet been elucidated.

The chemokine system is a critical aspect of the immune response that controls the migration of leukocytes into the brain. During infection chemokines are expressed and specific subsets of leukocytes migrate from blood into the brain. The importance of chemokines in controlling WNV infection is exemplified by the fact that chemokine CXC motif receptor 3 (CXCR3) and C-C chemokine receptor type 5 (CCR5) knock-out mice cannot clear infection and result in increased mortality. There is a clear need to define and understand the relative importance of the molecules that control leukocyte entry into the brain. For instance, it has been shown that TNF-α increases the vascular permeability and allows penetration of leukocytes in the perivascular space.

Once in the perivascular space, immune cells need to pass the second barrier, the glia limitans. Few studies have focused on understanding the molecular and cellular mechanisms regulating leukocyte penetration through the glia limitans. Expression of the C-X-C motif chemokine 12 (CXCL12) at the perivascular space is probably responsible for retention of cells in the perivascular spaces of the CNS [64]. In this regard, neutralization of CXCL12 was shown to promote leukocyte entry into the brain parenchyma [65]. On the other hand, matrix metalloproteinases (MMP), produced both by monocytes and glia cells, have been shown to be involved in migration of leukocytes to the perivascular space as well as migration through the glia limitans [66].

A study performed by Verma *et al.* using HBMVE and HBCA, showed that several MMPs were significantly induced in WNV-infected HBCA cells [45]. Incubation of naïve HBMVE cells with the supernatant from WNV-infected HBCA cells resulted in loss of tight junctions. These data provided evidence that astrocytes represent a source of MMP in the brain, which may lead to disruption of the BBB. Degradation of components of the glia limitans is another mechanism facilitating migration of leukocytes into the brain parenchyma. Collagen (a component of glia limitans) could be degraded by extracellular proteases such as the cysteine protease cathepsins K, S, and L [67,68], whereas conversion of plasminogen into plasmin may lead to degradation of laminin or fibronectin, other important components of the glia limitans [67,69]. When the integrity of the BBB is compromised, immune cells may enter the brain, thereby contributing to WNV viral clearance and immune mediated damage.

## 5. Pathogenesis of WNV Neuroinvasive Disease: A Balance between Viral Cytopathology and the Immune System

### 5.1. Mechanisms of Cell Death

Programmed cell death can be considered as a defense mechanism of the host in response to pathogenic insults. Pathogens may induce cell death to the host either by direct infection of host cells (e.g., cytolytic viruses) or by releasing toxic products (e.g., bacterial toxins). Cell death has been generally divided in necrosis (accidental, uncontrolled cell death resulting in inflammatory response) and programmed cell death, a regulated and controlled process, which traditionally has been considered non-inflammatory. Even though programmed cell death is often used as a synonym for apoptosis, it is more accurately described as cell death that is dependent on genetically encoded signals or activities within the dying cell [70,71]. No cellular activity is required as acute cell breakdown occurs as a result of the direct action of a damaging stimulus, and programmed cell death is therefore only prevented by the absence of this stimulus [70]. Recent studies have revealed several pathways that lead to programmed cell death: apoptosis, pyroptosis, autophagy and oncosis [72]. During viral infection, programmed cell death has an antiviral effect by inducing the death of infected cells. However, cell death can also have pathological effects if it occurs in non-renewing cell populations, such as neurons.

Apoptosis is mediated by a subset of cysteine-dependent aspartate specific proteases, or caspases, which can be divided into two functional subgroups: initiator caspases (caspase-2, -8, -9, and -10) are mainly involved in activation of the effector caspases-3, -6, and -7, which cleave a variety of cellular substrates. Apoptosis involves nuclear and cytoplasmic condensation and formation of membrane-bound cellular fragments or apoptotic bodies.

Pyroptosis is the proinflammatory pathway that results from caspase-1 activity and leads to membrane breakdown and proinflammatory cytokine processing. This pathway is uniquely dependent on caspase-1 [73–77], which is not involved in apoptosis. Caspase-1 deficient cells are able to respond normally to most apoptotic signals [78]. An important function of caspase-1 is to process the proforms of the inflammatory cytokines, interleukin (IL)-1β and IL-18, into their active forms [79]. Caspase-1 activation or dependence has often been observed during cell death in the immune [80], central nervous [81,82], and cardiovascular systems [83,84].

Many studies have shown that WNV induces replication-dependent apoptosis *in vitro*, and it has been hypothesized that virus-induced apoptosis contributes to neuronal death and the pathogenesis of encephalic flaviviruses [48,85–87]. Experiments in mice have been performed to analyze the occurrence of apoptosis *in vivo*. Most studies used the terminal deoxynucleotidyltransferase-mediated dUTP-biotin nick end labeling (TUNEL) staining, which detects DNA fragmentation, to confirm that apoptosis occurred in the CNS of WNV-infected mice.

Recently, evidence has been provided that the cell death of WNV-infected neurons is caspase-3 dependent [88]. It was shown that WNV infection induced caspase-3 activation and apoptosis in the brains of wild-type mice, while congenic caspase-3−/− mice were more resistant to lethal WNV infection. It is interesting to note that no significant differences in the tissue viral burdens or the kinetics of viral spread were found, but decreased neuronal death was observed in the cerebral cortices, brain stems, and cerebella of caspase-3−/− mice. Consistently, primary neurons derived from the CNS of wild-type mice showed caspase-3 activation and induction of apoptosis after WNV infection, and treatment with caspase inhibitors resulted in a significant decrease in virus-induced cell death. Nonetheless, a deficiency in caspase-3 did not completely protect neurons from WNV-mediated death *in vitro* or *in vivo*, indicating that caspase-3-independent pathways also contribute to WNV pathogenesis. For example, it is possible that the activation of non-caspase proteases, such as calphain and cathepsin family proteins is also triggered during WNV infection [89,90].

Yang *et al.* showed that direct expression of the WNV capsid protein in the striatum of mouse brain or interskeletal muscle caused cell death and inflammation [87]. Similar effects were observed in cultured, SH-SY5Y neuroblastoma cells, which could eventually be attributed to capsid-induced apoptosis occurring via the mitochondrial pathway, involving caspase-9 and caspase-3 activation. These studies suggest a role for the capsid protein of WNV in viral pathogenesis through the induction of the apoptotic cascade. No role for pyroptosis in the pathogenesis of WNV has yet been described. However, WNV has been shown to induce necrosis *in vitro* in cells exposed to very high viral inocula [91]. More effort should be deployed to define the different cell death pathways involved in the pathogenesis of severe WNV neuroinvasive disease.

The function of caspase-12 in viral immunity has not received much attention. Previously, it was shown that caspase-12 plays a role in endoplasmic reticulum stress-induced apoptosis in response to amyloid toxicity [92]. Wang *et al.* found that caspase-12-deficient mice had greater mortality, higher viral burden in peripheral (serum, lung, spleen) and neural (brain, cerebellum, spinal cord) tissues, and defective type I interferon response after WNV challenge compared to wild-type mice [93]. *In vitro* studies of primary neurons and mouse embryonic fibroblasts further demonstrated that caspase-12 positively modulated the production of type I interferon by regulating E3 ubiquitin ligase TRIM25-mediated ubiquitination of RIG-1, which is a critical signaling event for the type I interferon response to WNV and other viral pathogens. Alternatively, high levels of WNV non-structural (e.g., WNV-NS2A, 2B, 4A, 4B) or glycoprotein (WNV-E) may result in endoplasmic reticulum (ER) stress and unfolded protein response induction, resulting in apoptosis [94].

### 5.2. Immunopathology

How much cell injury can be attributed to viral cytopathology and how much to the inflammatory response is not known. Infection of neurons with WNV leads to the induction of several cytokines and chemokines, which promote leukocyte invasion into the CNS and neuroinflammation [95,96]. However, the extent to which this inflammation contributes to disease pathology remains unclear. In particular, the relative contribution of neurons to inflammation is a subject of intensive research.

Recently, it has been shown that WNV induced the expression of IL-1β, -6, -8, and tumor necrosis factor (TNF)-α in human neuroblastoma SK-N-SH cells in a dose- and time-dependent manner, which coincided with an increase in virus-induced cell death [97]. Treating cells with anti-IL-1β or anti-TNF-α resulted in significant reduction of the neurotoxic effects of WNV. When naïve astrocytes were treated with UV-inactivated supernatant from WNV-infected SK-N-SH cells, expression of glial fibrillary acidic protein (GFAP) and key inflammatory cytokines were increased. These results suggest that neurons are one of the potential sources of pro-inflammatory cytokines in WNV-infected brain, and that pro-inflammatory mediators are one of the main factors driving WNV-induced neurotoxicity. Recent studies with Japanese encephalitis virus (JEV; a closely related neurotropic flavivirus) also support a role of TNF-α in cell death, as increased expression of TNF-α receptors in neurons directly resulted in the initiation of death cascade via tumor necrosis factor receptor type 1-associated DEATH domain protein [98].

In animal models of JEV there is some evidence that activation of microglial cells plays a role in pathogenesis of encephalitis through the action of pro-inflammatory mediators, which induce neuronal cell death [99]. Although reactive gliosis (activation of astrocytes and microglia) has been reported in WNV neuroinvasive disease and is considered a key pathogenic feature [100–102], the extent to which infection of glial cells contributes to WNV-induced neurological disease has never been fully investigated. It is believed that collateral damage is mediated by inflammatory factors that are either neurotoxic or attract leukocytes into the affected area, which results in a detrimental inflammatory milieu.

Studies performed in Toll-like receptor 3 (TLR3) knock-out mice indicated that reduced production of the pro-inflammatory cytokines TNF-α and IL-6 by microglia was associated with their inability to promote injury of neuron-like cells and primary mouse neurons, whereas wild-type microglia released inflammatory cytokines and induced neurotoxicity. Recent evidence is provided by observational studies suggesting that macrophages from young individuals can down regulate TLR3 following infection with WNV, whereas macrophages of the elderly cannot. Therefore, it has been hypothesized that failure to down regulate TLR3 in infected cells results in production of high levels of pro-inflammatory and vasculogenic cytokines [103].

Furthermore, a study conducted by van Marle *et al.* using fatal cases of human WNV encephalitis suggested that WNV infects both neurons and glia cells, and that infection of these cells, in particular astrocytes, contributed to neuronal death by releasing neurotoxic mediators [95]. This study also showed an induction of neuroinflammatory genes, where a subset of these genes was specifically induced by the capsid protein of WNV. Particularly CXCL10, IL-1β and indolamine-2′,3′-deoxygenase (IDO) were shown to be over-expressed in astrocytes *ex vivo* and *in vivo*. Interestingly, production of CXCL10 by astrocytes has also been implicated in the neuropathogenesis of other viral infections, such as human immunodeficiency virus [103–107]. Nonetheless, further studies are needed to define the genetic programs associated with neuroprotection or the neurotoxic action of glial cells during WNV infection.

Recently, a paradoxical role for neutrophils in WNV pathogenesis has been described. When Bai *et al.* investigated the role of chemokines in WNV pathogenesis by infecting macrophages from mice with WNV, they found that expression of CXCL1 and CXCL2, which are two CXC-type chemokines that induce the migration of neutrophils, was dramatically up-regulated [108]. In addition, neutrophils were found to be the most abundant cell type in the peritoneal cavity as early as 12 hours after WNV inoculation. These results suggest that neutrophils are the predominant immune cells that are initially and rapidly recruited to sites of infection with WNV.

Interestingly, however, mice depleted of neutrophils had significantly lower WNV in their blood on day 2 or 3 after infection, and increased survival rates were seen. In contrast, when mice were infected with WNV before the depletion of neutrophils on days 1 and 2 after infection, they showed higher levels of viral load as well as reduced survival rates. The authors concluded that WNV may replicate in neutrophils and increase WNV load in blood early in infection, but that in the later course of infection these cells contribute to the control of infection. Eventhough these results should be confirmed, it is tempting to speculate that neutrophils play a critical role in WNV replication and dissemination *in vivo*, especially in humans where neutrophils are the predominant cell type in blood.

## 6. Concluding Remarks

Most studies conducted have elucidated a protective role of the immune system in WNV infection. Most of our knowledge regarding the immune response and pathogenesis has been derived from mouse studies. How the responses in birds differ from mammals and how it influences the course of disease has not been completely elucidated. Some studies suggest that differences in host response between different bird species may influence the outcome of WNV infection [109].

So far, only few studies have been able to demonstrate a pathogenic role for the immune system in inbred mice. A role has been implicated for TNF-α in modulating BBB permeability, and allowing dissemination of WNV into the brain where it can infect neurons and possibly glial cells. Direct infection of neurons may result in apoptosis via caspase-3 and -9. Furthermore, neurons also respond to WNV infection by up-regulating pro-inflammatory cytokines such as IL-1β, -6, -8 and TNF-α, which also contribute to neuronal damage. Infection of glial cells leads to up-regulation of CXCL10, IL-1β and IDO, and together with expression of MMP by infected astrocytes result in loss of tight junction and increased BBB permeability.

The role of the immune system in pathogenesis of WNV remains speculative. More studies need to be done to uncover further evidence for this hypothesis. Reasons why WNV-associated neuroinvasive disease is seen in less than 1% of individuals remain unexplained. However, it is plausible that after infection, WNV enters the brain of significantly more patients, but that certain host factors, such as an overactive inflammatory response, lead to excessive permeability of the BBB, as well as excessive neuronal death, eventually overruling the protective effects of the immune system.
